# Kinetics of naturally induced binding and neutralising anti-SARS-CoV-2 antibody levels and potencies among SARS-CoV-2 infected Kenyans with diverse grades of COVID-19 severity: an observational study

**DOI:** 10.12688/wellcomeopenres.19414.2

**Published:** 2024-12-02

**Authors:** John Kimotho, Yiakon Sein, Shahin Sayed, Reena Shah, Kennedy Mwai, Mansoor Saleh, Perpetual Wanjiku, Jedidah Mwacharo, James Nyagwange, Henry Karanja, Bernadette Kutima, John N. Gitonga, Daisy Mugo, Ann Karanu, Linda Moranga, Viviane Oluoch, Jasmit Shah, Julius Mutiso, Alfred Mburu, Zaitun Nneka, Peter Betti, Wanzila Usyu Mutinda, Abdirahman Issak Abdi, Philip Bejon, Lynette Isabella Ochola-Oyier, George M.Warimwe, Eunice W. Nduati, Francis M. Ndungu

**Affiliations:** 1 KEMRI-Wellcome Trust Research Programme, KILIFI, Coast, 230-80108, Kenya; 2 Pwani University, KILIFI, 230-80108, Kenya; 3 Aga Khan University Hospital, 3rd Parklands Avenue, Nairobi, 30270 - 00100, Kenya; 4 Centre for Tropical Medicine and Global Health, Nuffield Department of Medicine, University of Oxford, Oxford, UK; 5 Division of Infectious Diseases, Department of Medicine Solna and Center for Molecular Medicine, Karolinska Institutet, Stockholm, Sweden

**Keywords:** COVID-19, SARS-CoV-2, natural infection, binding-antibodies, neutralizing antibodies, kinetics, Kenya, sub-Saharan Africa

## Abstract

**Background:**

Given the low levels of coronavirus disease 2019 (COVID-19) vaccine coverage in sub-Saharan Africa (sSA), despite high levels of natural severe acute respiratory syndrome coronavirus-2 (SARS-CoV-2) exposures, strategies for extending the breadth and longevity of naturally acquired immunity are warranted. Designing such strategies will require a good understanding of naturally acquired immunity.

**Methods:**

We measured whole-spike immunoglobulin G (IgG) and spike-receptor binding domain (RBD) total immunoglobulins (Igs) on 585 plasma samples collected longitudinally over five successive time points within six months of COVID-19 diagnosis in 309 COVID-19 patients. We measured antibody-neutralising potency against the wild-type (Wuhan) SARS-CoV-2 pseudovirus in a subset of 51 patients over three successive time points. Binding and neutralising antibody levels and potencies were then tested for correlations with COVID-19 severities.

**Results:**

Rates of seroconversion increased from day 0 (day of PCR testing) to day 180 (six months) (63.6% to 100 %) and (69.3 % to 97%) for anti-spike-IgG and anti-spike-RBD binding Igs, respectively. Levels of these binding antibodies peaked at day 28 (p<0.01) and were subsequently maintained for six months without significant decay (p>0.99). Similarly, antibody-neutralising potencies peaked at day 28 (p<0.01) but declined by three-fold, six months after COVID-19 diagnosis (p<0.01). Binding antibody levels were highly correlated with neutralising antibody potencies at all the time points analysed (r>0.60, p<0.01). Levels and potencies of binding and neutralising antibodies increased with disease severity.

**Conclusions:**

Most COVID-19 patients generated SARS-CoV-2 specific binding antibodies that remained stable in the first six months of infection. However, the respective neutralising antibodies decayed three-fold by month-six of COVID-19 diagnosis suggesting that they are short-lived, consistent with what has been observed elsewhere in the world. Thus, regular vaccination boosters are required to sustain the high levels of anti-SARS-CoV-2 naturally acquired neutralising antibody potencies in our population.

## Introduction

Despite highly effective severe acute respiratory syndrome coronavirus-2 (SARS-CoV-2) vaccines, transmission and emergence of variants of concern continues ^1– 4^. Our understanding of the roles and durability of vaccine- and infection-induced anti-SARS-CoV-2 antibody responses in immunity to COVID-19 (coronavirus disease 2019) continues to evolve ^5– 7^. Antibodies, both infection and vaccine-induced are the most well-established correlates of protection against most of the medically important pathogens ^8, 9^. Neutralising antibodies against SARS-CoV-2 have also been suggested to be a possible correlate of protection against COVID-19 ^10– 14^. Antibodies offer protection against viruses through a myriad of mechanisms such as neutralisation, opsonisation, formation of immune complexes, complement deposition, and antibody-dependent cellular cytotoxicity ^15– 17^. SARS-CoV-2 virus infection elicits robust immunoglobulin G (IgG), immunoglobulin M (IgM), and immunoglobulin A (IgA) antibody responses targeting various epitopes of the virus. However, only a subset of these elicited antibodies has a neutralisation function ^18– 22^.

Neutralising antibodies offer protection by binding to viral epitopes, thereby interfering with virus attachment to the host cell receptor ^23^. The receptor binding domain (RBD), which is part of the SARS-CoV-2 spike protein, is the target of >90% of neutralising antibodies, which provides protection by blocking the virus from fusing with the angiotensin-converting enzyme (ACE2) of host cells ^20, 24– 26^.

Some of the previous studies in high-income countries have shown that naturally induced anti-SARS-CoV-2 antibodies could be short-lived ^26– 34^ while others reported the contrary ^6, 35– 48^. In addition, some COVID-19 patients from these countries have been shown to remain seronegative for SARS-CoV-2 antibodies despite being positive by RT-PCR (real-time-reverse transcription-polymerase chain reaction) ^39, 49, 50^, with some initially seropositive, reported to have sero-reverted ^26, 45, 51, 52^. Together, such data suggest the existence of inter-population differences in people’s abilities to generate and maintain anti-SARS-CoV-2 antibodies. However, there is a paucity of data on the kinetics and functions of anti-SARS-CoV-2 antibodies from sub-Saharan African populations ^53– 56^.

COVID-19 vaccine coverage in sub-Saharan Africa remains critically low ^57– 60^, with only about 31% of the African population having completed the initial two-dose vaccination protocol as of 20 ^th^ November 2024 ^59, 60^. Kenya has also faced low vaccine coverage ^61^ with approximately 63% of the adult Kenyan population (aged 18 years and above) and 90% of children (aged 12 years to below 18 years), remaining unvaccinated with at least two doses, as of February 2023 ^62^. This presented a unique window to study naturally acquired immunity to SARS-CoV-2 in our context, as the majority of the population has gained immunity primarily through natural infection rather than vaccination ^63– 67^. Comparing anti-SARS-CoV-2 immune responses between patients with different clinical phenotypes and determining the presence and longevity of antibody neutralisation function, may begin to provide clues for potential correlates of COVID-19 disease severity. This is particularly critical given the significantly lower rates of severe disease and mortality observed in sub-Saharan Africa compared to Europe and North America ^68– 72^.

Here, we describe the kinetics of naturally acquired anti-SARS-CoV-2 binding and virus-neutralising antibodies during the first six months of COVID-19 infection in a cohort of Kenyan patients with varying degrees of disease severity. To the best of our knowledge, this is the first longitudinal study evaluating the kinetics of naturally acquired anti–SARS-CoV-2 antibodies for a period that goes up to 6 months post-COVID-19 diagnosis, in a sub-Saharan African population.

## Methods

### Study sites

This study recruited patients over a period between June 2020 and February 2022 from Aga Khan University Hospital, Nairobi, an urban metropolitan academic medical centre. COVID-19 patients were recruited at hospitalisation, upon confirmation of SARS-CoV-2 infection by RT-PCR. A few of these patients who presented to the hospital for non-COVID-19 treatments and had no COVID-19 symptoms were also recruited as asymptomatic controls. We also recruited patients from Kilifi County Hospital, a community-based government hospital serving a rural coastal region. The Kilifi COVID-19 patients comprised mainly of outpatients with mild COVID-19 symptoms and their social contacts with asymptomatic SARS-CoV-2 infections, identified through COVID-19 surveillance.

### Ethical approvals

This study received approval from the Kenya Medical Research Institute’s Scientific and Ethics Review Unit (KEMRI SERU; protocol no. 4081), and the Aga Khan University, Nairobi, Institutional Ethics Review Committee (protocol no. 2020 IERC-135 V2).

### Study design

This was part of an ongoing longitudinal cohort study that aimed at understanding naturally acquired immune responses to SARS-CoV-2 among COVID-19 patients. Thus, the analysis excluded plasma samples obtained following COVID-19 vaccination of individual patients, as vaccines were introduced during the longitudinal follow-up. Written informed consent for participation in the collection of samples for the research, usage of those samples, and associated data, was offered to the participants following a positive diagnosis of COVID-19 with RT-PCR SARS-CoV-2 testing. Upon consenting, individual patients had a 10 mL venous blood sample taken using heparinised vacutainers, which formed the sampling baseline hereafter referred to as day 0. Subsequently, longitudinal follow-up venous 10 mL blood samples were taken on days 7, 14, 28, and 180 (6 months) from the positive COVID-19 test. Once a hospitalized patient had been discharged, they were reminded by telephone of their upcoming follow-up appointments and requested to visit the hospital for sample retrieval. At each of these times, plasma was immediately separated from the cells by centrifuging at 440g for 10 minutes using an Eppendorf 5810R centrifuge, and then aliquoted in microcentrifuge tubes which were then stored in -80°C freezers until the time of antibody measurements. Clinical data from medical record files were manually entered into REDCap electronic database and utilized to describe the baseline characteristics of the patients.

### Grading COVID-19 severity

Grading of COVID-19 presentations was done according to the National Institute of Health (NIH) ^73^ treatment guidelines as highlighted in Table 1. Clinical data were collected during patient treatment Extended data ^74^, and two-three doctors used it to define COVID-19 severities.

**Table 1.** Clinical Spectrum of SARS-CoV-2 Infection.

Grading COVID-19 severity	
Clinical Grade	Clinical manifestation
Asymptomatic or presymptomatic infection	Individuals who test positive for SARS-CoV-2 using a virologic test (i.e., a nucleic acid amplification test [NAAT] or an antigen test) but have no symptoms consistent with COVID-19.
Mild illness	Individuals who have any of the various signs and symptoms of COVID-19 (e.g., fever, cough, sore throat, malaise, headache, muscle pain, nausea, vomiting, diarrhea, loss of taste and smell) but do not have shortness of breath, dyspnea, or abnormal chest imaging.
Moderate illness	Individuals who show evidence of lower respiratory disease during clinical assessment or imaging and who have an oxygen saturation measured by pulse oximetry (SpO2 ) ≥94% on room air at sea level.
Severe illness	Individuals who have an SpO2 <94% on room air at sea level, a ratio of arterial partial pressure of oxygen to fraction of inspired oxygen (PaO2 /FiO2 ) <300 mm Hg, a respiratory rate >30 breaths/min, or lung infiltrates >50%.
Critical illness	Individuals who have respiratory failure, septic shock, or multiple organ dysfunction.

### Anti-spike IgG antibody ELISA

Anti-spike IgG antibodies were measured using a previously described Enzyme-linked immunosorbent assay (ELISA) developed and validated at KWTRP, Kilifi, Kenya with a sensitivity of 92.7% (95% CI 87.9–96.1%) and specificity of 99.0% (95% CI 98.1–99.5%) targeting the full-length trimeric spike ^64^. Briefly, 2 μg/ml of whole trimeric spike protein was coated in Nunc MaxiSorp™ flat-bottom 96-well plates (ThermoFisherScientific) at 37 °C for one hour. The plates were then washed three times in wash buffer (0.1% Tween 20 in 1X phosphate buffered saline- 1X PBST) and blocked with Blocker™ Casein (ThermoFisherScientific) for one hour at room temperature. Heat-inactivated plasma samples were diluted 1:800 in Blocker™ Casein, added to the plates, and incubated for two hours at room temperature. After three further washes, 100 μl horseradish peroxidase (HRP)-conjugated goat antihuman IgG antibody (Catalogue number 074–1002, KPL-SeraCare), diluted 1:10, 000 in wash buffer, was added to the plates and incubated for one hour at room temperature. After three washes, the plates were developed with o-phenylenediamine dihydrochloride (OPD) substrate (Sigma) for 10 min and read on an Infinite® 200 PRO microplate reader (TECAN) at 492 nm. The results were expressed as the ratio of test optical density (OD) to the OD of the plate negative control; samples with OD ratios > 2 were considered positive for SARS-CoV-2 IgG and those with OD ratios ≤ 2 as negative. To estimate the antibody levels, we used an international standard ^75^ of pooled human plasma obtained from COVID-19 convalescent patients.

### Anti-RBD (IgA, IgG, IgM) ELISA

We used a commercial receptor binding domain (RBD) total antibody ELISA kit (MABTECH AB, Nacka strand, Sweden, Lot 3890-1H-R1-1) and followed the manufacturer’s instructions to detect RBD-spike binding Igs of the isotypes (IgA, IgM, and IgG). This kit has a specificity: negative percent agreement (NPA) of 100% (51/51 un-infected controls) and a sensitivity: positive percent agreement (PPA) of 100% (72/72 COVID-19 convalescents - PCR confirmed). Briefly, 96-well plates were coated overnight at 4 °C with 100µl per/well of strep-tactin® XT diluted at 1µg/ml. The coating solution was then discarded, and blocking was done using 200µl of the dilution buffer per well for 1 hour. Washing was then done five times using 300µl per well of wash buffer. Plasma was diluted at 1:64 using the dilution buffer and 50µl was added in duplicate to the plates followed by 50µl per well of RBD Bridge reconstituted at 1:12. The plates were then placed on an orbital plate shaker at 400 revolutions per minute for 2 hours at room temperature (RT). The plates were then washed five times. Anti-Wiskott-Aldrich syndrome protein - horseradish peroxidase peptide tag (Anti-WASP-HRP) was diluted 200 times and was added at 100µl per well to the plates and incubation was done for 1 hour at RT. Washing was then done 10 times and 100µl per well of Tetra-methyl benzidine (TMB) was added. Plates were then incubated for 15 minutes in the dark at RT before adding 100µl per well of stop solution. Absorbance was measured at 450nm using a BioTek Synergy H1 Multimode Reader. To quantify the antibody levels, hyperimmune plasma with a top standard dilution at 1:650 was assigned an arbitrary 1000 ELISA units and diluted serially, 2-fold. Therefore, anti-spike-RBD Igs data were presented as arbitrary ELISA units (AEU). A four-parameter logistic model generated by Gene5™ 2.0 analytical software was used to determine the arbitrary concentrations of the antibodies. 10 pre-COVID-19 negative control plasma samples that were available at the KEMRI Wellcome Trust Research Program (KWTRP) sample Biobank, were included in the assay to establish a cut-off of - seropositivity calculated at six standard deviations of the mean of negative controls, according to the manufacturer’s instructions.

### SARS CoV-2 pseudovirus production and neutralisation assay

Pseudoviruses were generated by co-transfecting murine leukaemia virus-gag/pol (MLV-gag/pol), murine leukemia virus-cytomegalovirus-Luciferase (MLV-CMV-Luciferase) and SARS-CoV-2 Wuhan-1 spike plasmids with Polyethylenimine Max (PEI MAX®) (Polysciences) into human embryonic kidney 293T/17 cells (HEK293T/17). Culture supernatants were clarified of cells (after 72 hours) by 0.45μM filter and stored at -80°C. HeLa-human angiotensin converting enzyme 2 cells (HeLa-hACE2), modified to overexpress human ACE2, were cultured in Dulbecco's Modified Eagle Medium (DMEM) with GlutaMAX ^TM^ (Gibco-BRL) containing 10% heat-inactivated foetal calf serum (HIFCS) (Sigma-Aldrich) and 1% of 100x Penicillin-Streptomycin (Pen-Strep) (Sigma-Aldrich) at 37°C, 5% Carbon dioxide (CO _2_). At confluency, cell monolayers were disrupted using 0.25% trypsin-1 mM disodium ethylenediaminetetraacetic acid (EDTA) (Gibco-BRL). Plasma samples were heat-inactivated, clarified by centrifugation, and serially diluted in 96-well flat-bottom culture plates (Corning, 3595) containing growth medium. Pseudoviruses and serially diluted plasma were incubated for 1 hour at 37°C, 5% CO _2_. HeLa-hACE2 cells were added at 10,000 cells per well and incubated for 72 hours at 37°C, 5% CO _2_. Supernatants were removed, the cells were lysed in Glo lysis buffer 1X (Promega, E2661), luciferase activity was measured by adding Bright-Glo (Promega, E2650) and luminescence was measured using BioTek Synergy H1 multimode reader. Neutralisation was measured as described by a reduction in luciferase gene expression after a single round infection of Hela-hACE2 cells with spike-pseudo typed viruses. Levels were calculated as the reciprocal plasma inhibitory dilution (ID _50_) causing a 50% reduction in relative light units (RLU).

### Statistics

Comparisons of antibody levels across time points were performed using Friedman’s test for repeated measures, followed by pairwise Wilcoxon signed-rank tests for post hoc multiple comparisons. LOWESS regression curves were generated to depict the overall trajectories of antibodies levels over time. Spearman’s correlation coefficients were calculated to assess the relationships between RBD-specific immunoglobulins, anti-spike IgG, and neutralization ID50 antibody levels at each time point. Associations between disease severity and antibody responses, as well as the effects of time, age, and gender, were assessed using a linear mixed model. Kruskal-Walli’s test, followed by Dunn’s multiple comparison tests, was used to compare antibody levels across different clinical severity groups. The Mann-Whitney U test was applied for non-parametric pairwise comparisons. Analyses were performed using R V4.1.2 (RRID: SCR_001905) and some graphs were visualized using GraphPad Prism V8.0.2. (RRID: SCR_002798). A P value of < 0.05 was considered statistically significant.

## Results

### COVID-19 cohort recruitment

**Figure 1.**
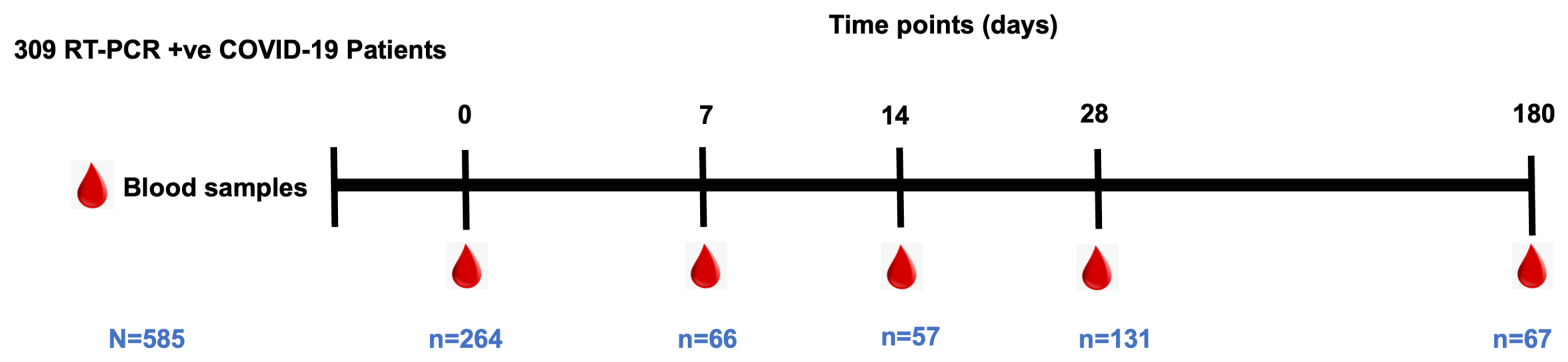
Longitudinal blood sampling over six months of Coronavirus disease 2019 (COVID-19 diagnosis). Indicates the total number of plasma samples collected at each time point.

We recruited a total of 309 COVID-19 patients, from whom a total of 585 blood samples had been collected across the five-time points described here ( Figure 1). From AKUH, Nairobi, we recruited a total of 270 hospitalised COVID-19 patients between June 2020 to September 2021, and from KCH, Kilifi, we recruited 39 COVID-19 patients between March 2021 to February 2022. Not every patient was available for all their scheduled times for follow-up samples. Thus, whilst 49.5% (153/309) of the patients had samples available from multiple time points, the rest only contributed to samples at day 0. To address the random missing data, individuals with only a single time point sample were excluded from statistical analyses involving repeated measurements, such as linear mixed modeling of longitudinal data. All participants included in the neutralisation assay had a plasma sample available at all the three-time points assayed. However, the percentages of seropositive participants and calculations of median antibody levels at each time point were calculated based on the number of samples available.

### Clinical characterisation of the COVID-19 cohort

70% of the patients were male, and the median age increased with disease severity ( Table 2). Majority of patients were in the mild (39.2%, 121/309) and severe (45.6%, 141/309) groups. The remaining patients were distributed among the asymptomatic (6.8%, 21/309), moderate (7.4%, 23/309), and critical (0.9%, 3/309) categories. Underlying non-communicable diseases, including diabetes and hypertension, were common, particularly among patients with severe and critical illness. All critically ill patients (100%, 3/3) had both diabetes and hypertension and required intensive care unit (ICU) support. Among severe patients, 56% (79/141) had diabetes, 32.6% (46/141) were hypertensive, and 16.3% (23/141) required ICU care. In the moderately sick group, 26.1% (6/23) were diabetic, 13% (3/23) were hypertensive, and 4.3% (1/23) required ICU care. In the mild group, 37.2% (45/121) were diabetic, 22.3% (27/121) were hypertensive, with no patients requiring ICU admission. None of the asymptomatic participants had diabetes or hypertension.

**Table 2.** Patient’s baseline characteristics categorised according to the different grades of coronavirus disease 2019 (COVID-19) severities. Table generated using the “Tableone” package in R software. The continuous non-parametric variable is represented as a median with an interquartile range (IQR). Categorical variables are represented as frequencies. COPD-Chronic obstructive pulmonary disease, ICU- Intensive care unit.

	Asymptomatic	Mild	Moderate	Severe	Critical
Number	21	121	23	141	3
**Demographics**					
Age (median [IQR])	35.00 [29.00, 54.00]	46.29 [36.22, 54.47]	46.00 [35.50, 56.89]	52.21 [43.00, 59.48]	67.15 [60.06, 69.14]
Gender = Male (%)	12 ( 57.1)	77 ( 63.6)	16 ( 69.6)	108 ( 76.6)	2 ( 66.7)
= Female (%)	9 (42.9)	44 (36.4)	5 (30.4)	33 ( 23.4)	1 (33.3)
Race (%)					
African American / Black	21 (100.0)	106 ( 87.6)	20 ( 87.0)	125 ( 88.7)	3 (100.0)
Asian	0 ( 0.0)	6 ( 5.0)	2 ( 8.7)	5 ( 3.5)	0 ( 0.0)
Caucasian	0 ( 0.0)	5 ( 4.1)	1 ( 4.3)	4 ( 2.8)	0 ( 0.0)
Indian	0 ( 0.0)	4 ( 3.3)	0 ( 0.0)	7 ( 5.0)	0 ( 0.0)
**Comorbidities**					
Diabetes = Yes (%)	0 ( 0.0)	45 ( 37.2)	6 ( 26.1)	79 ( 56.0)	3 (100.0)
Hypertension = Yes (%)	0 ( 0.0)	27 ( 22.3)	3 ( 13.0)	46 ( 32.6)	3 (100.0)
HIV Positive = Yes (%)	0 ( 0.0)	5 ( 4.1)	0 ( 0.0)	5 ( 3.5)	1 ( 33.3)
Renal disease = Yes (%)	0 ( 0.0)	1 ( 0.8)	0 ( 0.0)	3 ( 2.1)	0 ( 0.0)
COPD = Yes (%)	0 ( 0.0)	0 ( 0.0)	0 ( 0.0)	1 ( 0.7)	0 ( 0.0)
Cancer = Yes (%)	0 ( 0.0)	0 ( 0.0)	0 ( 0.0)	2 ( 1.4)	0 ( 0.0)
**Patient ** **management (%)**					
Hospital admission= Yes (%)	0 ( 0.0)	103 ( 85.1)	23 (100.0)	141 (100.0)	3 (100.0)
In ICU = Yes (%)	0 ( 0.0)	0 ( 0.0)	1 ( 4.3)	23 ( 16.3)	3 (100.0)
**Outcome**					
Outcome = Died (%)	0 ( 0.0)	1 ( 0.83)	1 ( 4.3)	6 ( 4.3)	0 ( 0.0)

### Anti-SARS-CoV-2 binding antibody seroconversion rates and maintenance for the first six months of positive COVID-19 diagnosis

**Figure 2.**
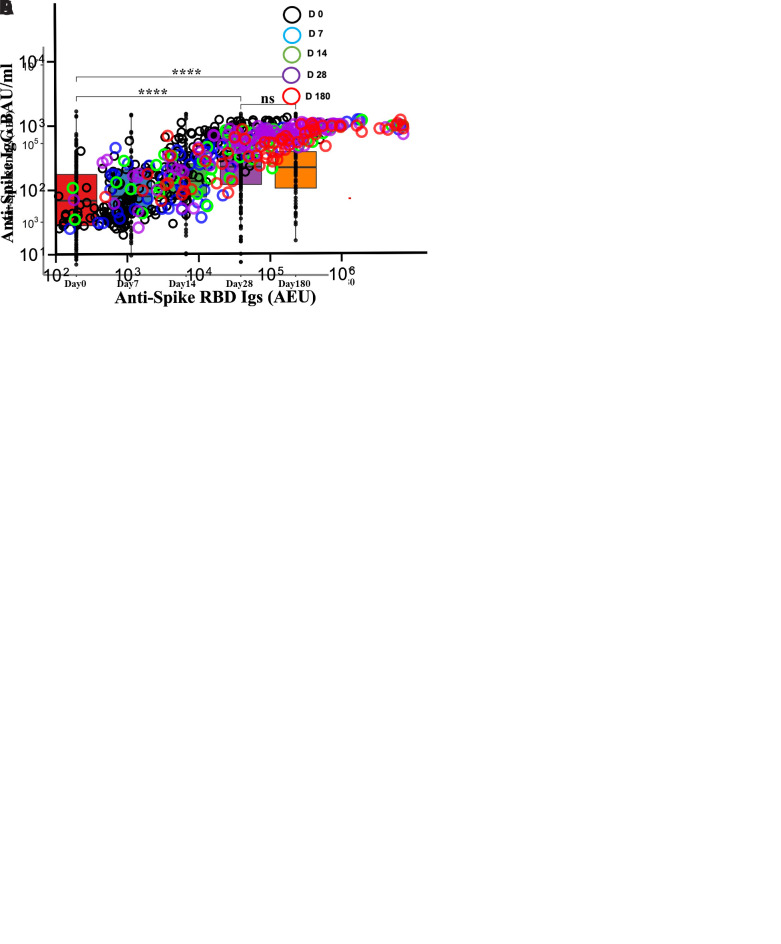
Antibody level kinetics, and correlation of anti-spike IgG and anti-spike-Receptor binding domain (RBD) immunoglobulins. ( **A**) Kinetics of anti-spike IgG and ( **C**) anti-spike-RBD Igs over the first six months of coronavirus disease 2019 (COVID-19) diagnosis. The dotted red line represents the threshold of sero-positivity defined by the negative controls as described in the methods. The blue curve depicts the best fit non-linear regression model of antibody trajectories. ( **C**) Box plots depicting anti-spike IgG antibody kinetics and anti-spike-RBD Igs ( **D**) over 6 months as calculated with the Friedman’s test of repeated measures with Pairwise Wilcoxon signed-rank test for multiple comparison correction. ( **E**) Depicts Spearman’s correlation of anti-Spike IgG binding antibody units (BAU) and anti-spike-RBD Igs arbitrary ELISA unit (AEU) levels at all time points. *P < 0.05, **P < 0.01, ***P < 0.001, ****P < 0.0001

Seroconversion rates for both antigens increased over time following COVID-19 diagnosis ( Table 3). At day 0, only 63.6% (168/264) of patients had seroconverted for spike IgG, whereas all patients with available samples at day 180 were antibody positive (67/67). For anti-spike-RBD Igs, 69.3% (183/264) of patients had seroconverted at day 0, and of those with multiple successive samples, 97% (65/67) remained antibody positive at day 180. Of the patients with plasma samples from more than two time points, 7.8% (12/155) exhibited a sharp increase in antibody levels from day 28 to day 180, suggesting possible re-infections ( Figure 2A & C).

**Table 3.** Severe acute respiratory syndrome (SARS-CoV-2) seroconversion rates. Depicts percentages of sero-positivity and negativity over time. Sero-conversion cut-offs were determined using the negative controls as described in the methods.

Anti-spike IgG			
Time point	Plasma samples	Sero-positivity = n (%)	Sero-negativity= n (%)
day 0	264	168 (63.6)	96 (36.4)
day 7	66	54 (81.8)	12 (18.2)
day 14	57	54 (94.7)	3 (5.3)
day 28	131	126 (96.2)	5 (3.8)
day 180	67	67 (100)	0(0.0)
Anti-spike-RBD Igs			
Time point	Plasma samples	Sero-positivity = n (%)	Sero-negativity = n (%)
day 0	264	183 (69.3)	81 (30.7)
day 7	66	55 (83.3)	11 (16.7)
day 14	57	51 (89.5)	6 (10.5)
day 28	131	121 (92.4)	10 (7.6)
day 180	67	65 (97.0)	2(3.0)

Anti-spike IgG levels increased from a median level of 171.6 BAU/ml (IQR, 46.00 - 553.8) from day 0 to 804.57 BAU/ml (IQR,550.37 - 1021.74) on day 28 (P<0.01, Friedman test with Pairwise Wilcoxon post hoc test) ( Figure 2B). These levels were thereafter maintained without significant decay with day 180 median levels for anti-spike IgG being 604.42 BAU/ml (IQR,312.58 - 933.05) (p >0.90). Overall, anti-spike IgG antibody BAU/ml levels increased with the time of COVID-19 diagnosis (Estimate = 0.36, P<0.01), after adjusting for gender and age, in a linear mixed model analysis of patients with at least more than two plasma samples (( Extended data ^74^, Table 1). When anti-spike-RBD Igs were considered, their levels increased from a median of 3510.38 AEU (IQR, 811.73 -16111.00) on day 0, to 25723.71 AEU (IQR, 9098.85 - 53537.56) on day 28 (P<0.01) and were also maintained without significant decay at day 180 with levels at 25002.75 AEU (IQR,7343.15 - 62111.00) (p >0.90) ( Figure 2D). The levels of anti-spike IgG and anti-spike-RBD Igs binding antibodies were strongly correlated with each other at all the time points: day 0 (r =0.84, P<0.01), day 7 (r =0.85, P<0.01), day 14 (r =0.84, P<0.01), day 28 (r =0.82, P<0.01) and day 180 (r =0.84, P<0.01), Spearman’s correlation coefficient) ( Figure 2E).

### Kinetics of neutralisation antibody levels over the first six months of COVID-19 diagnosis

We further investigated if the antibodies induced by SARS-CoV-2 infection could elicit viral neutralisation in a subset of 51 patients whose plasma samples were available at the time points of days 0, 28, and 180 post infection. The spread-out timepoints allowed for a more comprehensive analysis of neutralization kinetics. The 51 patients included 7 asymptomatic, 21 mild, 21 severe, 1 moderate, and 1 critically ill individual. Given the single participant in both the moderate and critical illness categories, the moderately ill patient was included in the mild group (due to more similar clinical presentation), while the critically ill patient was included in the severe group to ensure adequate statistical power for comparison. These 51 individuals had similar antibody kinetics to what was observed for the entire study as reported above and therefore representative of the study population ( Extended data ^74^, Figure 1).

**Figure 3.**
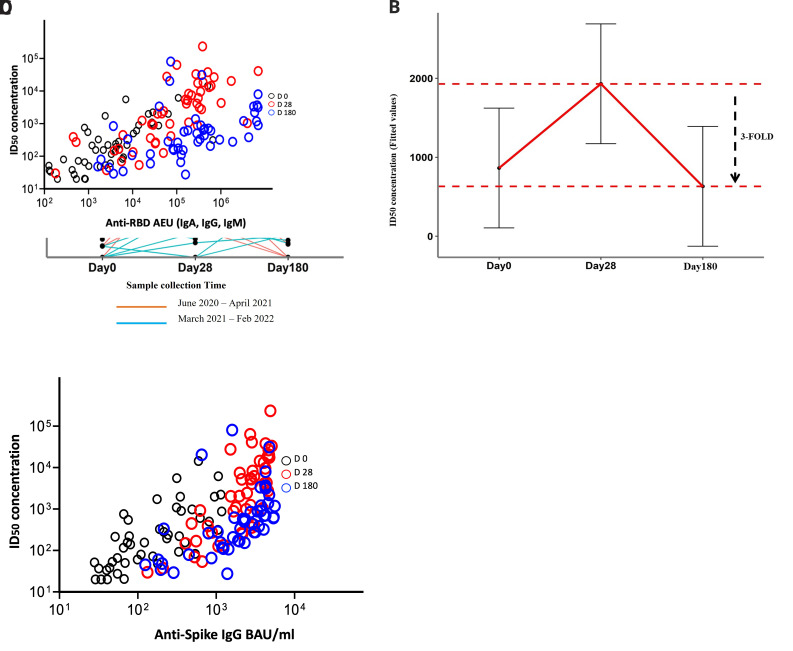
Neutralising inhibitory dilution (ID50) potencies, kinetics, and correlations with binding antibodies. This analysis includes 51 COVID-19 patients who had plasma samples available at the time points of days 0, 28, and 180. ( **A**) Kinetics of neutralising ID50 potencies over three-time points of coronavirus disease 2019 (COVID-19) diagnosis coloured according to the plasma sample collection time. Statistics were done with Friedman’s test for repeated measures with the Pairwise Wilcoxon signed-rank test for multiple comparison correction. ( **B**) Fitted values from a linear mixed model to depict the rate of decline in neutralising antibodies towards day 180. ( **C**) Spearman’s correlation between neutralising ID50 and anti-spike IgG and ( **D**) between neutralising ID50 and anti-spike-RBD Igs at the three-time points *P < 0.05, **P < 0.01, ***P < 0.001, ****P < 0.0001.

A total of 90.2% (46/51) of the patients had already developed neutralising antibodies by day 0. On day 28, the proportion of responders had increased to 96.1% (49/51), and it was 94% (48/51) at day 180. For all the participants, antibody neutralisation potency increased significantly from a median 50% inhibitory dilution (ID _50_) of 155.10 (IQR, 42.92 - 578.79) at day 0 and peaked at day 28 post-infection at a median ID _50 _of 621.88 (IQR, 145.89 - 1958.61) (P<0.01, Friedman test with Pairwise Wilcoxon post hoc test), before rapidly declining to a median ID _50 _of 153.00 (IQR, 66.07-326.08) at day 180 (P<0.01) ( Figure 3A). To quantify the decline, we fitted a linear mixed model describing the kinetics of neutralisation antibody levels ( Extended data ^74^, Table 2) and then extracted the fitted values to obtain independent estimates of neutralising antibody levels at each time point. When the independent estimate fitted values of ID _50 _1929.928 at day 28 were compared with those of ID _50_ 630.9376 at day 180, we found that the neutralising antibody levels had decreased by three-fold ( Figure 3B).

Interestingly, for those participants who had been recruited earlier in the pandemic (pre-April 2021), the trajectory of antibody neutralisation levels depicted an increase towards day 28 post-infection followed by a decline towards day 180 ( Figure 3A). However, for those recruited later in the pandemic, (post-April 2021), the responses were more heterogenous, with neutralising antibody levels increasing over time and plateauing between day 28 and day 180.

To establish if the magnitude of the neutralisation antibody potencies varied with changes in the circulating SARS-CoV-2 strains at the time of recruitment, we categorised participants based on available data in the public domain on the SARS-CoV-2 strains that were circulating in Kenya circulating at the time of patient sampling ^76, 77^, as we did not have the sequence data to ascertain the precise strain infecting the patients. On this basis, participants were categorised into two groups, those recruited earlier in the pandemic and hence likely to have been infected with the Wuhan strain, and those recruited later, and hence likely to have been infected by other SARS-CoV-2 variants like alpha, beta, delta, and omicron.

We observed higher neutralising antibody potency from the day 0 samples of those recruited before April 2021 with a median ID _50 _of 222.06 (IQR, 92.73-874.11) as compared to those sampled after April 2021 with a median ID _50 _of 49.47 (IQR, 20.00-82.79) (P<0.01, Mann Whitney test) ( Extended data ^74^, Figure 2). At day 28, pre-April 2021 plasma samples maintained higher neutralising antibody potency with median ID _50_ 1287.73 (521.54 - 3059.15) as compared to post-April 2021 plasma samples ID _50 _129.83 (IQR, 56.04 - 356.96) (P<0.01). At day 180, both groups exhibited similar neutralizing antibody potencies, with median ID50 values of 151.02 (IQR, 41.55 – 218.77) for the pre-April 2021 cohort and 168.93 (IQR, 75.56 – 408.75) for the post-April 2021 cohort (p=0.38).

When antibody neutralisation potencies were correlated with the binding antibody levels, high correlations were observed: for the IgG antibodies against the whole spike protein, r =0.81 (P<0.01, Spearman’s correlation coefficient) at day 0, r=0.77 (P<0.01) at day 28, and r =0.75 (P<0.01) at day 180 ( Figure 3C), and for the spike-RBD Igs, r =0.79 (P<0.01) at day 0, r =0.80 (P<0.01) at day 28, and r =0.60 (P<0.01) at day 180 ( Figure 3D).

### Associations of spike binding and virus-neutralising antibody potencies with COVID-19 severity

**Figure 4.**
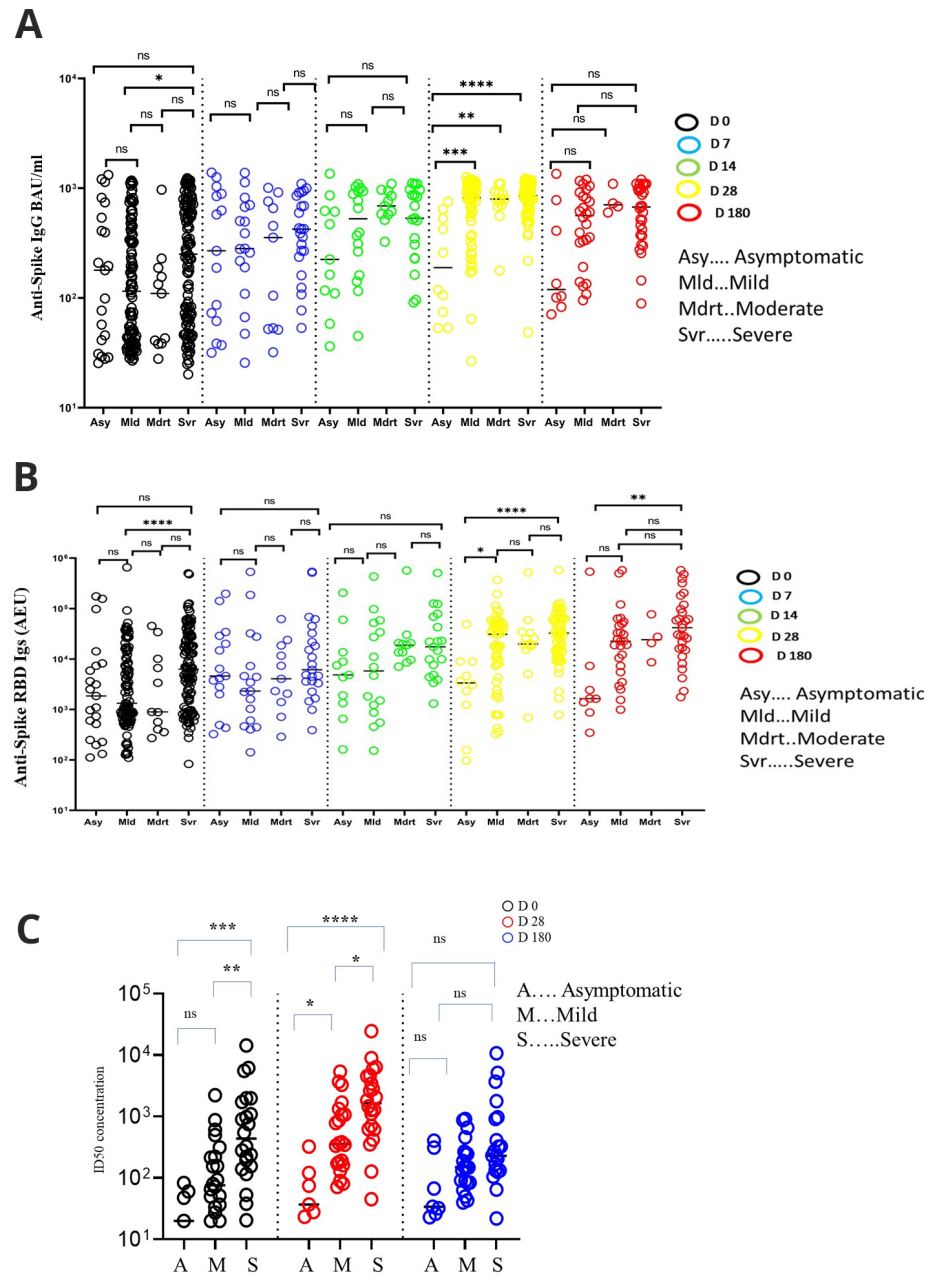
Comparisons of antibody levels across different grades of COVID-19 severities. Schematic depicting comparisons of antibody levels among clinical groups, anti-spike IgG ( **A**) and anti-spike-RBD Igs ( **B**). ( **C**) A comparison of neutralising inhibitory dilution (ID50) potencies across disease phenotypes at each of the three-time points. All comparisons were done using Kruskal-Walli’s method with Dunn’s multiple comparison correction. * P < 0.05, **P < 0.01, ***P < 0.001, ****P < 0.0001.

Increasing levels of binding antibodies were positively associated with disease severity with asymptomatic and severe patients having the lowest, and the highest antibody levels respectively, at earlier time points of the infection ( Figure 4A and 4B). For instance, day 0 anti-spike IgG levels were higher among the severe than the mild group, with median levels of 250.90 (IQR, 64.37 - 700.00) and 115.20 (IQR, 40.41 - 406.5) BAU/ml, respectively (p = 0.02, Kruskal Wallis test with Dunn’s test for multiple comparisons). Similarly, day 28 anti-spike IgG antibody levels were higher among the severe than the asymptomatic group, with median levels of 855.80 (IQR, 615.00 - 1099.00) and 189.10 (IQR, 69.23 - 559.60) BAU/ml, respectively (P<0.01). Anti-spike IgG antibodies on day 28 were also higher among the mild than the asymptomatic, with median levels of 821.10 (IQR, 407.50 - 1024) and 189.10 (IQR, 69.23 - 559.60), respectively (P<0.01). The moderate group had higher anti-spike IgG antibody levels than the asymptomatic group, with median levels of 797.7 (IQR, 690.80 - 916.2) and 189.10 (IQR, 69.23 - 559.60) BAU/ml, respectively (p=0.03). This positive association between increasing anti-IgG levels and disease severity remained significant in a linear mixed model, even after adjusting for gender and age (Estimate= 0.18, P<0.01, Extended data ^74^, Table 1). However, on day 180, these antibodies were all similar in all the pairwise group comparisons.

Day 0 neutralising antibody potencies were higher among severe than mild (P=0.01) and asymptomatic individuals (P<0.01), with medians ID _50_ being 436.50 (IQR, 151.70 - 1795.00) 76.04 (IQR, 31.96 - 244.90) and 20.00 (IQR, 0.00 - 60.64), respectively ( Figure 4C). At day 28, severe patients maintained higher neutralising antibody potencies with a median ID _50_ of 1653 (IQR, 621.70 - 4589.00) than the asymptomatic ID _50 _37.03 (IQR, 23.44 - 121.10) (P<0.01), and mild patients median ID _50 _357.00 (IQR, 153.90 - 1138.00) (p=0.03). The mild patients had higher neutralising antibody potencies than asymptomatic individuals (p= 0.04). However, the neutralisation antibody potencies had dropped significantly by day 180 and were no longer different in any of the pairwise group comparisons.

### Associations of viral loads with COVID-19 Severity

Because differences in viral loads could explain the associations between antibody levels and functions with disease severity, we compared viral loads among groups using cycle threshold (Ct) values from the day 0 RT-PCR SARS-CoV2. The comparisons within each of the two hospitals were done separately as different RT-PCR assays had been used. This was done for a total of 189 patients (asymptomatic =13, mild=67, moderate= 17, severe= 92), for whom viral-load data were available. Patients who had missing Ct values had been referred from other health facilities and had a positive COVID-19 diagnosis made from those health facilities, but their Ct values were unavailable. We found no evidence that the viral loads were associated with the different clinical phenotypes ( Extended data ^74^, Figure 3). However, we recognise the limitation of small numbers in some of the groups.

## Discussion

Most of the COVID-19 patients in this study seroconverted and maintained SARS-CoV-2 spike binding antibodies for the first six months after positive COVID-19 diagnosis, without significant decay. In a subset of the individuals, antibodies with virus-neutralising function against a Wuhan SARS-CoV-2 strain-based pseudovirus were observed, which peaked 28 days after COVID-19 diagnosis. However, neutralising antibody potency had dropped three-fold by six months from COVID-19 diagnosis. Furthermore, the ability to neutralise the pseudovirus was higher in plasma samples collected during the period when the Wuhan strain was spreading in Kenya and then diminished, but not completely abolished, in those collected later in the pandemic when the subsequent SARS-CoV-2 variants of concern were the most prevalent strains. Both the antibody-binding levels and neutralising potencies were strongly correlated and increased with disease severity. The study’s longitudinal design allowed us to evaluate the kinetics of naturally acquired anti–SARS-CoV-2 antibodies over a 6-month period following COVID-19 diagnosis. This is particularly significant as it provides unique insights into the durability of binding and neutralising antibody responses in a sub-Saharan African population, a demographic that has been underrepresented in immunological research on SARS-CoV-2.

The observation that binding antibody levels were stable for the first six months of COVID-19 diagnosis, is consistent with some of the previous reports in populations from high-income countries (HIC), where anti-SARS-CoV-2 antibodies were maintained for the first few months of infection ^6, 35– 48^. However, other studies from HIC populations found binding antibodies to wane quickly within the first few months after infection ^26– 34^. Very few studies have determined the kinetics of anti-SARS-CoV-2 antibody kinetics in sub-Saharan African populations ^53– 56^. A study from Ethiopia followed PCR-confirmed COVID-19 patients for a median time of 31 days used lateral flow immunoassays to quantify anti-spike and anti-nucleoprotein IgM, IgG, and an electro-chemiluminescent assay to quantify total anti-nucleoprotein immunoglobulins. They reported heterogeneous antibody kinetics and a lack of seroconversion among some patients. A South African study reported rapid waning of anti-SARS-CoV-2 RBD IgA and IgM, but better maintenance of IgG antibodies quantified weekly for 3 months among COVID-19 patients ^78^. Our results suggest longer maintenance of anti-SARS-CoV-2 antibodies and highlight the importance of longer durations of follow-ups in longitudinal studies ascertaining antibody kinetics following infection.

Our finding, that neutralising antibodies decayed three-fold by the sixth month of follow-up, suggests that anti-SARS-CoV-2 antibodies with neutralising function are short-lived. This finding is in agreement with previous studies in other populations, which also found neutralising antibodies to significantly decline after a few months of infection ^29, 30, 41, 79– 81^. Some studies have however reported anti-SARS-CoV-2 neutralising antibodies to persist for more than six months ^36, 37, 44, 82– 84^. The decline of neutralizing antibodies is a significant concern, as neutralisation is one of the most critical immune defenses against viral infections. This probably explains possible re-infections in our study, where a few patients had binding and neutralising antibodies increasing after day 28 of COVID-19 diagnosis (without vaccination). Cases of possible SARS-CoV-2 re-infections have also been reported elsewhere ^85– 88^.

The decreasing ability of plasma antibodies to neutralise the Wuhan-based strain pseudovirus with the time of sampling probably reflects the subsequent gradual replacement of the Wuhan strain with others like alpha, beta, delta, and omicron in Kenya ^76, 77^. We however note that did not have the sequence data to ascertain the precise strain infecting the patients and so we utilised data available in the public domain on SARS-CoV-2 strains that were circulating in Kenya circulating at the time of patient sampling. Better neutralisation potencies to the infecting strain and reduced neutralisation to alternative stains has been confirmed elsewhere. For example, recognitions of B.1.1.7- Alpha and B.1.351-Beta strains were reduced in antibodies elicited by the parental Wuhan and D614G strains ^89– 91^. Nonetheless, the fact that we could still detect antibodies with neutralisation potency against the Wuhan strain-based pseudovirus throughout the six months of follow-up in this longitudinal study indicates a good level of cross-reactivity across the different variants as reported elsewhere ^92, 93^. Further assessment of cross-neutralisation among different variants will be important in understanding the extent of infection-induced immunity in protecting from future SARS-CoV-2 variants during periods of high transmission, as well as the effectiveness of the current SARS-CoV-2 vaccines. Therefore, it will be important to determine the neutralising breadth of antibodies generated against the parental Wuhan virus and COVID-19 immunisations, against upcoming SARS-CoV-2 variants of concern.

Both spike-binding antibody levels and neutralisation potencies increased with COVID-19 severity, especially so in the first month of diagnosis. Similar findings have been reported elsewhere, where anti-spike and anti-spike-RBD antibody concentrations were found to be higher in severely ill COVID-19 patients compared to mildly ill patients during the acute stages of infection ^94, 95^. Of note, a study on Kenyan COVID-19 patients, found symptomatic COVID-19 patients to have higher spike IgG antibodies as compared to asymptomatic patients ^63^. Neutralising antibodies were also higher among severely than mildly sick USA COVID-19 patients ^96^, and ICU patients compared to non-ICU Chinese patients ^97^ and have not necessarily correlated with better disease outcomes during primary infection ^41, 98, 99^. Despite the clear finding of higher binding antibody levels and neutralising function among the severe than mild and asymptomatic patients, the causal effect relationship remains unclear. Higher viral loads, inflammation, or both, could explain the induction of higher antibody levels and neutralisation potencies. However, our study did not find differences in viral loads among the disease severities, perhaps owing to the small sample size in that analysis.

## Conclusions

Our results suggest that SARS-CoV-2 neutralising antibodies are short-lived, whilst other antibodies may be long-lasting. Hence, binding antibodies are not an accurate surrogate of neutralising function, especially during convalescence as has also been observed elsewhere ^42^. Aside from neutralisation, antibodies can also mediate viral clearance through other mechanisms via their fragment crystallisable (Fc) region, such as antibody-dependent cellular cytotoxicity and complement deposition. However, such roles have not been as investigated as compared to those of neutralisation functions SARS-CoV-2 ^100– 102^. However, virus neutralisation is one of the main mechanisms by which COVID-19 vaccines work ^103– 105^ and may be more likely to offer sterile immunity compared to other antibody-mediated mechanisms. Understanding antibody kinetics, both binding affinities and functions will be important in informing current vaccination strategies, and in the development of second-generation COVID-19 vaccines that may be more likely to offer sterile immunity and much-needed long-term protection. In the meantime, efforts to broaden and extend the current levels of naturally acquired anti-SARS-CoV-2 immunity with vaccination followed by regular administrations of regular vaccine boosters will be required to sustain the high levels of neutralising antibodies that peak after acute infection.

## Abbreviations

SARS-CoV-2: severe acute respiratory syndrome coronavirus 2; COVID-19: Coronavirus disease 2019; AEU: Arbitrary ELISA units; BAU: Binding antibody units; Ct: Cycle threshold; ELISA: Enzyme-linked immunosorbent assay; HRP: Horseradish peroxidase; Immunoglobulin G; IgM: Immunoglobulin M, IgA: Immunoglobulin A; Ig: Immunoglobulin; ID _50_: 50% inhibitory dilution OD: Optical density; OPD: o-Phenylenediamine; PBS: Phosphate buffered saline; Room temperature; RT-PCR: Reverse transcription polymerase chain reaction; RBD: Receptor binding domain; RLUs: Relative light units; WASP-HRP: Wiskott-Aldrich syndrome protein - Horseradish peroxidase.

## Data Availability

Harvard Dataverse: Replication Data for: Kinetics of naturally induced binding and neutralizing anti-SARS-CoV-2 antibody levels and potencies among Kenyan patients with diverse grades of COVID-19 severity. https://doi.org/10.7910/DVN/6KW9U1 ^74^ This project contains the following underlying data: Baseline_characteristics_Binding_antibody_data.tab Merged_Neutralization_work.tab ImmunoCoV_Clinical_Data.tab Harvard Dataverse: Replication Data for: Kinetics of naturally induced binding and neutralizing anti-SARS-CoV-2 antibody levels and potencies among Kenyan patients with diverse grades of COVID-19 severity. https://doi.org/10.7910/DVN/6KW9U1 ^74^ This project contains the following extended data: ImmunoCov_Supplementary_Material.pdf GraphPad_ImmunoCoV_MS_codes.pzfx ImmunoCoV_Codes_Markdown.pdf JKimotho_ImmunoCoV_Readme.txt JKimotho_ImmunoCoV_Data_Dictionary.xlsx Data are available under the terms of the Creative Commons Attribution 4.0 International license (CC-BY 4.0).
