# Supplementary material for: Kinetics of naturally induced binding and neutralising anti-SARS-CoV-2 antibody levels and potencies among SARS-CoV-2 infected Kenyans with diverse grades of COVID-19 severity: an observational study
Source: Wellcome Open Res. 2024 Dec 2;8:350. Originally published 2023 Aug 17. [Version 2] doi: 10.12688/wellcomeopenres.19414.2 (PMC11617823; doi:10.12688/wellcomeopenres.19414.2)
Supplement: Peer review history

# Reviewer report: Reviewer response for version 2

**DOI:** 10.21956/wellcomeopenres.25890.r114115

**Published:** 2024-12-04

**Authors:** Eleonora Nicolai

**Affiliations:** 1 University of Tor Vergata, Rome, Italy

Authors addressed all concerns arisen in the previous revision. I find the present article interesting and clear and I suggest for indexing.

Is the work clearly and accurately presented and does it cite the current literature?

Yes

If applicable, is the statistical analysis and its interpretation appropriate?

Yes

Are all the source data underlying the results available to ensure full reproducibility?

Yes

Is the study design appropriate and is the work technically sound?

Yes

Are the conclusions drawn adequately supported by the results?

Yes

Are sufficient details of methods and analysis provided to allow replication by others?

Yes

Reviewer Expertise:

biophysics and biochemistry

I confirm that I have read this submission and believe that I have an appropriate level of expertise to confirm that it is of an acceptable scientific standard.

# Reviewer report: Reviewer response for version 2

**DOI:** 10.21956/wellcomeopenres.25890.r114114

**Published:** 2024-12-04

**Authors:** Flaminia Tomassetti

**Affiliations:** 1 University of Rome Tor Vergata, Rome, Italy

The authors have adequately addressed the comments made by the reviewers in the revised version of the manuscript. Therefore, I have no further comments.

Is the work clearly and accurately presented and does it cite the current literature?

Partly

If applicable, is the statistical analysis and its interpretation appropriate?

Yes

Are all the source data underlying the results available to ensure full reproducibility?

Yes

Is the study design appropriate and is the work technically sound?

Yes

Are the conclusions drawn adequately supported by the results?

Yes

Are sufficient details of methods and analysis provided to allow replication by others?

Yes

Reviewer Expertise:

Clinical Biochemistry, Pathology, Immunology

I confirm that I have read this submission and believe that I have an appropriate level of expertise to confirm that it is of an acceptable scientific standard.

# Reviewer report: Reviewer response for version 1

**DOI:** 10.21956/wellcomeopenres.21508.r103687

**Published:** 2024-11-01

**Authors:** Flaminia Tomassetti

**Affiliations:** 1 University of Rome Tor Vergata, Rome, Italy

The manuscript "Kinetics of naturally induced binding and neutralising anti-SARS-CoV-2 antibody levels and potencies among SARS-CoV-2 infected Kenyans with diverse grades of COVID-19 severity: an observational study" seems quite interesting and has the potential to add a valuable piece to the knowledge of SARS-CoV-2 immunity and antibody kinetics.

 The article is well-written and structured correctly, the results are expressed concisely and are supported by figures and tables, the discussion is critical and bears the data found, therefore, I recommend minor revision for this work.

**Minor revision:**

**Abstract:**

 Clear and well written

**Introduction:**

 The Authors summarize efficiently the state of art of the SARS-CoV-2 spread, immunity and epidemiology and the aim is concise and understandable. I only suggest updating some references to more recent studies.

**Material and methods:**

 I suggest to include a table for the paragraph "Grading COVID-19 severity" to a better stratification of the population studied.

**Result:**

- The Authors should decide in which form to express the decimals (two decimals after the point or three)
- The information of *r* and *p*, in the Figure 2a, 3c and 3d results a little bit chaotic; the Authors should delete them in the figure and move the information in the description.

**Discussion:**

 The authors should include the strengths and the limitation of their work.

Is the work clearly and accurately presented and does it cite the current literature?

Partly

If applicable, is the statistical analysis and its interpretation appropriate?

Yes

Are all the source data underlying the results available to ensure full reproducibility?

Yes

Is the study design appropriate and is the work technically sound?

Yes

Are the conclusions drawn adequately supported by the results?

Yes

Are sufficient details of methods and analysis provided to allow replication by others?

Yes

Reviewer Expertise:

Clinical Biochemistry, Pathology, Immunology

I confirm that I have read this submission and believe that I have an appropriate level of expertise to confirm that it is of an acceptable scientific standard, however I have significant reservations, as outlined above.

# Response

**Published:** 2024-11-20

**Authors:** John Kimotho

**Affiliations:** Bioscience, KEMRI Wellcome Trust Research Program, Kilifi, Coast, Kenya

**Introduction:**

**The Authors summarize efficiently the state of art of the SARS-CoV-2 spread, immunity and epidemiology and the aim is concise and understandable. I only suggest updating some references to more recent studies.** 

***Response:*** *Thank you for highlighting that. We have reviewed more recent literature and updated the introduction as suggested.*

**Material and methods:**

**I suggest to include a table for the paragraph "Grading COVID-19 severity" to a better stratification of the population studied.**

***Response:*** *This has been effected. A table has been included as a summary. Thank you for that suggestion.*

**Result:**

**The Authors should decide in which form to express the decimals (two decimals after the point or three).** **·         The information of r and p, in the Figure 2a, 3c and 3d results a little bit chaotic; the Authors should delete them in the figure and move the information in the description.**

***Response:*** *We have decided to stick to two decimal places for consistency. The information of r and p has been removed from the figures and moved to the text.*

**Discussion:**

**The authors should include the strengths and the limitation of their work.**

***Response:*** *This has been effected in the discussion, thank you for highlighting that.*

# Reviewer report: Reviewer response for version 1

**DOI:** 10.21956/wellcomeopenres.21508.r82159

**Published:** 2024-06-08

**Authors:** Eleonora Nicolai

**Affiliations:** 1 University of Tor Vergata, Rome, Italy

Dear Editor,

 the paper by Kimotho and co-workers describes the kinetics of naturally acquired anti-SARS-CoV-2 binding and virus neutralizing antibodies during the first six-month od CPVID-19 infection in a cohort of Kenyan patients with varying degrees of disease severity.

 Authors results suggest that SARS-CoV-2 neutralizing antibodies are short-lived, while other antibodies may be long-lasting.

 I found the present work interesting in order to gain useful information for regularization of boosters doses.

 I have only some minor concerns.

 Pag 5: I didn't get the meaning of the last sentence of the paragraph "Anti-spike IgG antibody ELISA". Please check and eventually rephrase.

 Pag 6: in the first line of the paragraph "Anti-SRS-CoV-2 binding antibody seroconversion rates and maintenance for the ......" there is a typo error in Igs instead of IgS.

 Figure 2: for the sake of clarity I suggest to put panel A in the high and middle part of the figure,

 panel B and D below and panel C and E below.

 Pag 12: authors underline the importance of longer durations of follow-ups. Few lines below they state that only few studies have reported anti-SARS-CoV-2 neutralizing antibodies to persist for more than six months. And the report only 2 references. I suggest to author to deepen they literature research on this item.

Is the work clearly and accurately presented and does it cite the current literature?

Yes

If applicable, is the statistical analysis and its interpretation appropriate?

Yes

Are all the source data underlying the results available to ensure full reproducibility?

Yes

Is the study design appropriate and is the work technically sound?

Yes

Are the conclusions drawn adequately supported by the results?

Yes

Are sufficient details of methods and analysis provided to allow replication by others?

Yes

Reviewer Expertise:

biophysics and biochemistry

I confirm that I have read this submission and believe that I have an appropriate level of expertise to confirm that it is of an acceptable scientific standard, however I have significant reservations, as outlined above.

# Response

**Published:** 2024-11-20

**Authors:** John Kimotho

**Affiliations:** Bioscience, KEMRI Wellcome Trust Research Program, Kilifi, Coast, Kenya

**Pag 5: I didn't get the meaning of the last sentence of the paragraph "Anti-spike IgG antibody ELISA". Please check and eventually rephrase.**

***Response:*** *This has been rephrased. We meant that we used a WHO recommended standard in the anti-spike IgG ELISA. It is made of pooled human plasma with an assigned concentration. It had been obtained from COVID-19 convalescent patients.*

**Pag 6: in the first line of the paragraph "Anti-SRS-CoV-2 binding antibody seroconversion rates and maintenance for the ......" there is a typo error in Igs instead of IgS.**

***Response:*** *We used Igs “in plural” as an abbreviation of immunoglobulins throughout the manuscript, which we wrote in full in the first mention. This is because we were referring to three types of immunoglobulins i.e. anti-spike-RBD IgA, IgG and IgM hence “anti-spike-RBD Igs”.*

**Figure 2: for the sake of clarity I suggest to put panel A in the high and middle part of the figure,**

**panel B and D below and panel C and E below.** *This has been corrected as suggested,* ***Response:*** *thank you for that observation.*

**Pag 12: authors underline the importance of longer durations of follow-ups. Few lines below they state that only few studies have reported anti-SARS-CoV-2 neutralizing antibodies to persist for more than six months. And the report only 2 references. I suggest to author to deepen they literature research on this item.**

***Response:*** *We have reviewed more literature on the subject and identified a total of 6 references-some recent -supporting long-term persitence of anti-SARS-CoV-2 neutralizing antibodies for more than six months.*
